# Distinct microglia alternative splicing in Alzheimer's disease

**DOI:** 10.18632/aging.204223

**Published:** 2022-08-23

**Authors:** Yanjun Lu, Lu Tan, Jiazhao Xie, Liming Cheng, Xiong Wang

**Affiliations:** 1 Department of Laboratory Medicine, Tongji Hospital, Tongji Medical College, Huazhong University of Science and Technology, Wuhan 430030, China; 2 Key Laboratory for Molecular Diagnosis of Hubei Province, The Central Hospital of Wuhan, Tongji Medical College, Huazhong University of Science and Technology, Wuhan 430014, China; 3 Department of Pathophysiology, Key Laboratory of Ministry of Education for Neurological Disorders, School of Basic Medicine, Tongji Medical College, Huazhong University of Science and Technology, Wuhan 430030, China

**Keywords:** alternative splicing, Alzheimer's disease, microglia, skipped exon, retained intron

## Abstract

Numerous alternative splicing (AS) events have been documented in Alzheimer's disease (AD). However, cell type-specific AS analysis is still lacking. We described AS events in the hippocampal microglia sorted by CD45 and CD11b from Aβ precursor protein (APP) and non-transgenic (Ntg) mice. GSE171195 dataset was downloaded from GEO database, aligned to GRCm39 genome. Skipped exon (SE), alternative 3’SS (A3SS), retained intron (RI), alternative 5’SS (A5SS), and mutually exclusive exons (MXE) were evaluated using rMATS and maser. Differential expressed genes or transcripts were analyzed via limma. Gene ontology and correlation analyses were performed with clusterProfiler and ggcorrplot R packages. 36,340 raw counts of AS were identified, and 95 significant AS events were eventually selected with strict criteria: (1) average coverage >5; (2) delta percent spliced in >0.1. SE was the most common AS events (68.42%), followed by A3SS and RI. Autophagy genes were mainly spliced in SE events, actin depolymerization genes spliced in A3SS events, while synaptic plasticity related genes were mainly spliced in RI pattern. These significant AS events may be regulated by dysregulated splicing factors in AD. In conclusion, we revealed microglia specific AS events in AD, and our study provides novel pathological mechanisms in the pathogenesis of AD.

## INTRODUCTION

Splicing converts precursor mRNA into mature mRNA via spliceosome, a multiprotein-RNA complex with highly specific and stepwise interactions. The spliceosome contains 5 small nuclear ribonucleoproteins (snRNPs; U1 to U6 snRNPs). U1 snRNP binds to 5′ splice site, and U2 snRNP binds to 3′ splice site and polypyrimidine sequences. Next, U5, U4/U6 were recruited to complete intron excision and exon linkage mainly following the “GU-AG” rule [1]. Alternative splicing (AS) enables cells to diversify their proteome by generating distinct characteristics to different protein isoforms through different combination of exons in the mRNA, leading to complex biological functions under conditions of external and internal stimuli. AS plays essential roles in cell differentiation and organ development t [2]. Generally, five basic modes of AS are classified: alternative 5′ splice site (A5SS), skipped exon (SE), mutually exclusive exons (MXE), retained intron (RI), and alternative 3′ splice site (A3SS) [3]. AS dynamically changes during aging, and aberrant AS events have been observed in numerous diseases including cancer through providing malignant protein isoforms [4]. Carcinogenesis involves cellular proliferation, invasion and metastasis, induction of angiogenesis, and immune escape. Recurrent somatic mutations of the splicing machinery components have been observed in human solid tumors and hematological malignancies. AS participates in these processes by regulating the alternative expression of numerous oncogenic or tumor suppressor genes [5].

Aberrant AS events have been characterized in Alzheimer’s disease (AD), the most frequent cause of dementia. AD is hallmarked by β-amyloid (Aβ) plaques and hyperphosphorylated tau tangles. Both genetic and environmental risk factors contribute to AD pathogenesis [6]. The triggering receptor expressed in myeloid cells 2 (TREM2) mediates inflammatory responses, microglia activation, and phagocytosis of apoptotic neurons. TREM2 is involved in tau accumulation and Aβ plaque formation and therefore strongly affects risk of AD [7]. TREM2 rare variants caused exon 2 skipping, and activated immune-related functional pathways in AD [8]. Nuclear speckle specific hnRNP D-like prevented AD-related cognitive decline via modulating AS of synaptic gene calmodulin kinase-like vesicle-associated (CAMKV) [9]. Numerous AS events mainly including SE and RI have been documented in both AD human and animal models [10, 11].

These studies mainly focused on different models or brain regions. We systematically described AS events in the hippocampal microglia (CD45int CD11b+) sorted with FACSAria II (BD) of Aβ precursor protein (APP) and non-transgenic (Ntg) mice. We revealed hippocampal microglia cell type-specific AS events in AD.

## RESULTS

### Global summary of alternative splicing events

**Figure 1:**
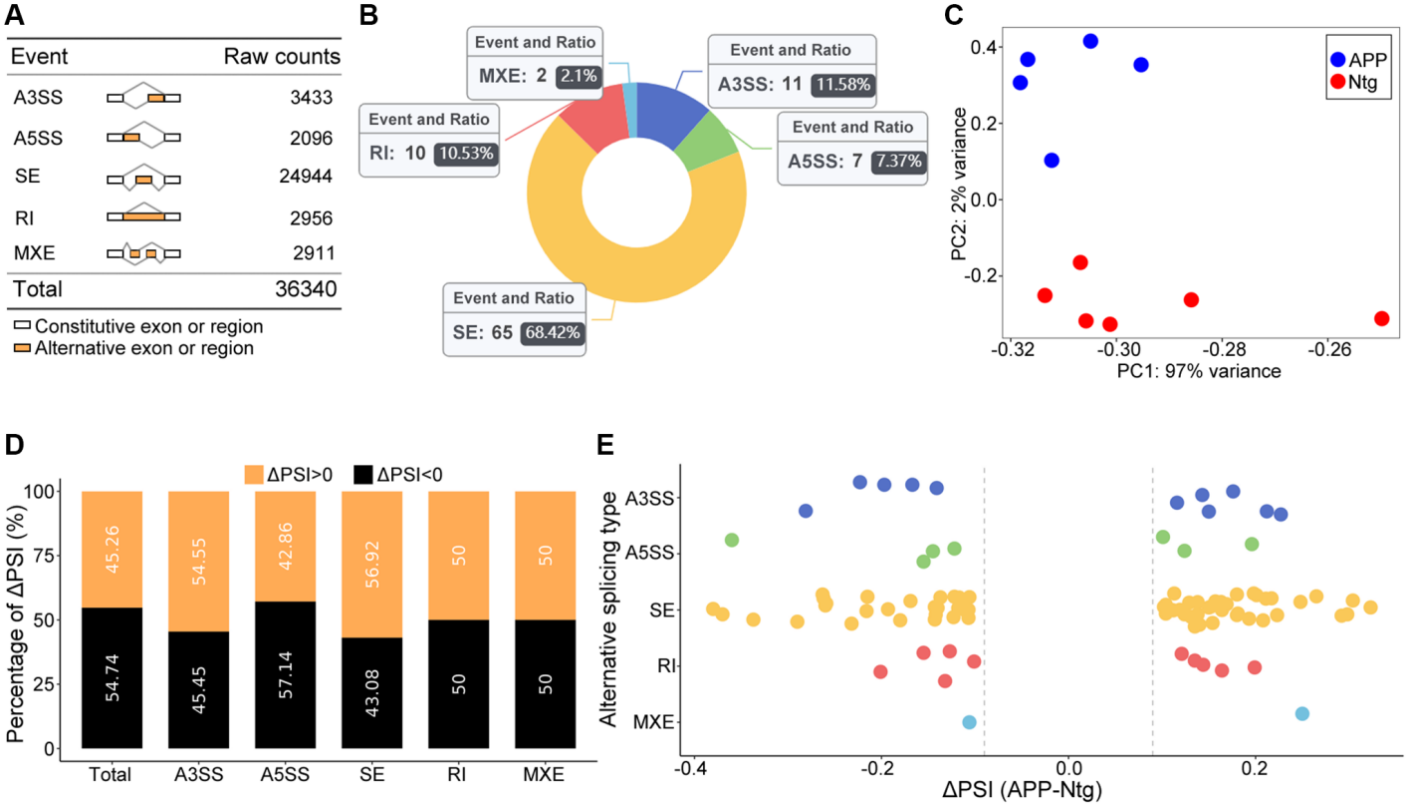
**Global summary of AS events.** (**A**) Raw counts in each AS event. (**B**) Number and ratio of each significant AS event. (**C**) The PCA plot based on PSI values. APP and Ntg groups were clearly classified. (**D**) The percent of ΔPSI in each and global AS events. (**E**) Distribution of ΔPSI in each type AS events.

A total of 36,340 AS events were initially identified in GSE171195 dataset (Figure 1A). Eventually, 95 significant AS events were collected (Figure 1B, Table 1). The principal component analysis (PCA) revealed that these AS events clearly classified APP from Ntg group (Figure 1C). Increased PSI was found in A3SS and SE in APP group, while decreased PSI was found in A3SS and global events (Figure 1D, 1E). Top two events in each AS event were included in Table 2, and all 95 significant AS events were shown in Supplementary Table 1.

**Table 1:** Event summary.

**Event type**	**Total events junction counts**	**Average coverage > 5**	**FDR < 0.05, deltaPSI > 0.1**	**Ratio in Ntg**	**Ratio in APP**
A3SS	3433	2413	11	11.60%	11.50%
A5SS	2096	1337	7	9.30%	5.80%
SE	24944	19866	65	65.10%	71.20%
RI	2956	2034	10	11.60%	9.60%
MXE	2911	2465	2	2.30%	1.90%
Total	36340	28115	95	100.00%	100.00%

**Table 2:** Top two events in each type of AS pattern.

**ID**	***P* value**	**FDR**	**ΔPSI**	**Gene**	**Type**
3991	1.39E-07	0.000238	0.15	Tamm41	A3SS
3756	2.60E-07	0.000297	−0.167	Ube2f	A3SS
33	2.02E-07	0.000211	−0.145	Incenp	A5SS
2020	2.34E-06	0.001637	0.101	Dcun1d2	A5SS
482	4.45E-09	1.23E-05	0.298	Rnls	SE
29753	1.63E-08	3.12E-05	0.304	Rcor3	SE
1605	2.91E-06	0.000782	−0.127	Rbm3	RI
3926	8.27E-05	0.009775	−0.101	Myl6	RI
3222	7.50E-11	2.18E-07	−0.106	Pld3	MXE
3301	6.08E-05	0.02213	0.25	Erbin	MXE

### Analysis of SE events

**Figure 2:**
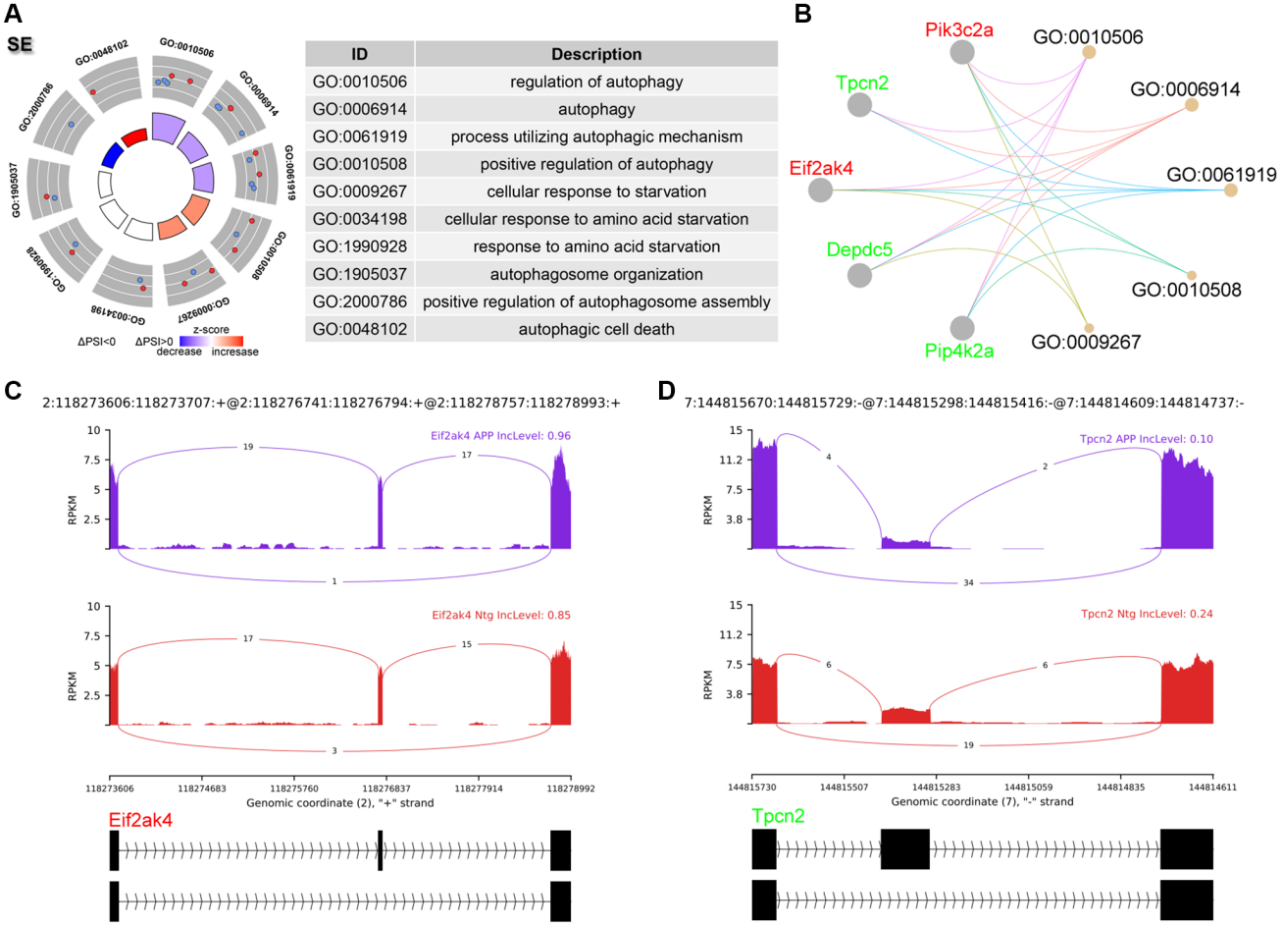
**Analysis of SE events.** (**A**) Significant GO terms enriched in genes involved in SE events. (**B**) Cnetplot revealed genes in these enriched GO terms. Eif2ak4 colored in red indicated increased PSI level, while Tpcn2 colored in green represented decreased PSI level. (**C**, **D**) The detailed sashimi plots for Eif2ak4 and Tpcn2.

SE events ranked the most frequent AS events, accounting for 68.42% (Figure 1B). GO analysis of genes involved in significant SE events were enriched in regulation of autophagy (Figure 2A, 2B), including eukaryotic translation initiation factor 2 alpha kinase 4 (Eif2ak4) and two pore segment channel 2 (Tpcn2). Eif2ak4 tended to harbor longer exons, while Tpcn2 tended to harbor shorter exons in APP (Figure 2C, 2D).

### Analysis of A3SS events

**Figure 3:**
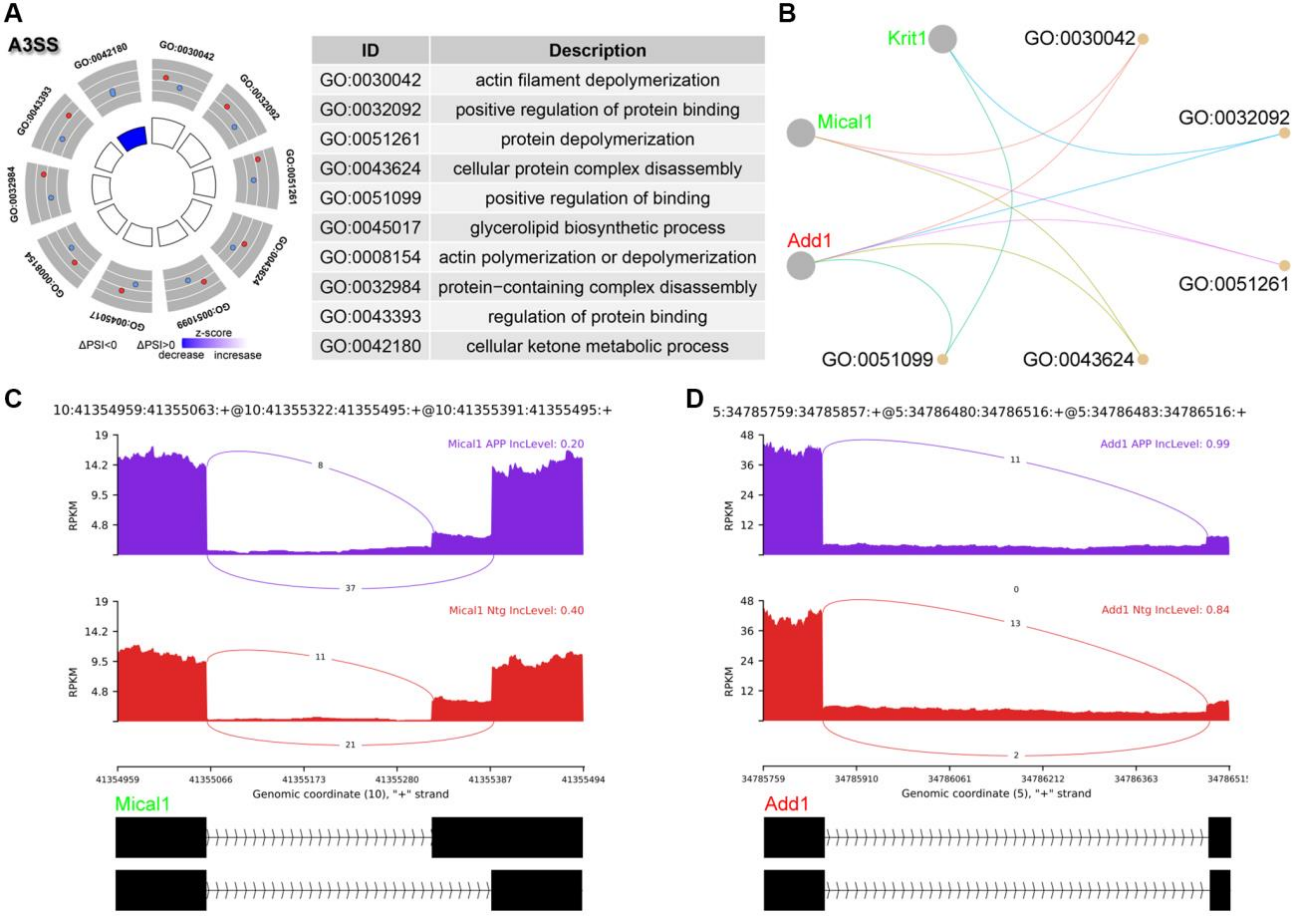
**Analysis of A3SS events.** (**A**) Significant GO terms enriched in genes involved in A3SS events. (**B**) Cnetplot revealed genes in these enriched GO terms. Mical1 colored in red indicated increased PSI level, while Add1 colored in green represented decreased PSI level. (**C**, **D**) The detailed sashimi plots for Mical1 and Add1.

A total of 11 significant A3SS events were identified and ranked top 2 AS events (Figure 1B). GO enrichment revealed that genes with significant A3SS events were enriched in actin polymerization and depolymerization, including microtubule associated monooxygenase, calponin and LIM domain containing 1 (Mical1), and adducin 1 (Add1) (Figure 3A, 3B). The sashimi plot showed decreased PSI and a shorter transcript for Mical1 (Figure 3C), whereas increased PSI for Add1, indicating a longer transcript (Figure 3D).

### Analysis of RI events

**Figure 4:**
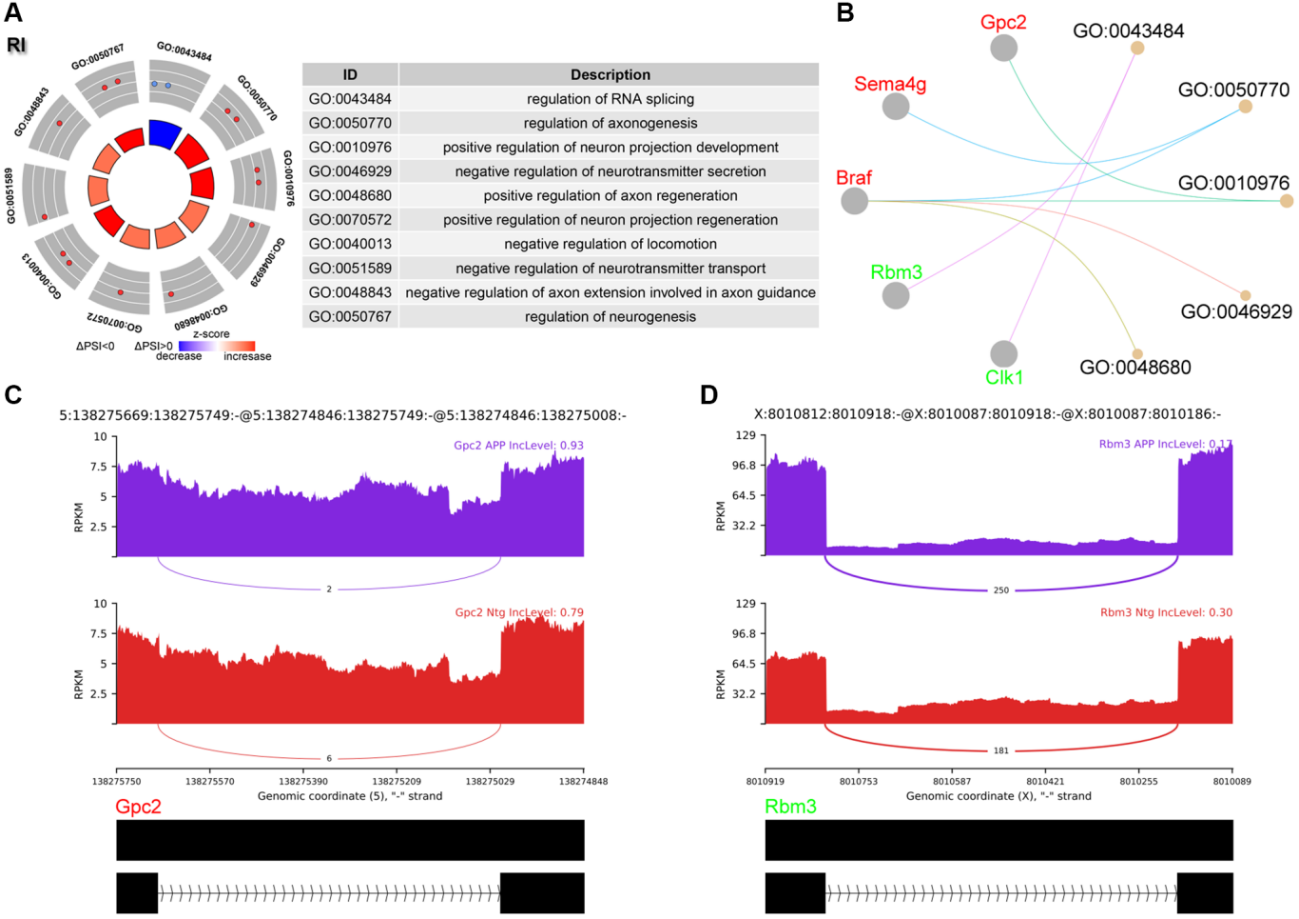
**Analysis of RI events.** (**A**) Significant GO terms enriched in genes involved in RI events. (**B**) Cnetplot revealed genes in these enriched GO terms. Gpc2 colored in red indicated increased PSI level, while Rbm3 colored in green represented decreased PSI level. (**C**, **D**) The detailed sashimi plots for Gpc2 and Rbm3.

RI is third most common events in this study (Figure 1B). The PSI value referred to level of retained intron. GO analysis revealed enrichment of RNA splicing as well as synaptic plasticity including axonogenesis, neurotransmitter secretion, neuron projection and axon guidance (Figure 4A, 4B). Glypican 2 (Gpc2) showed increased RI level and enriched in neuron projection (Figure 4C). RNA binding motif protein 3 (Rbm3) showed decreased RI level and enriched in RNA splicing (Figure 4D).

### Differentially expressed genes and transcript

**Figure 5:**
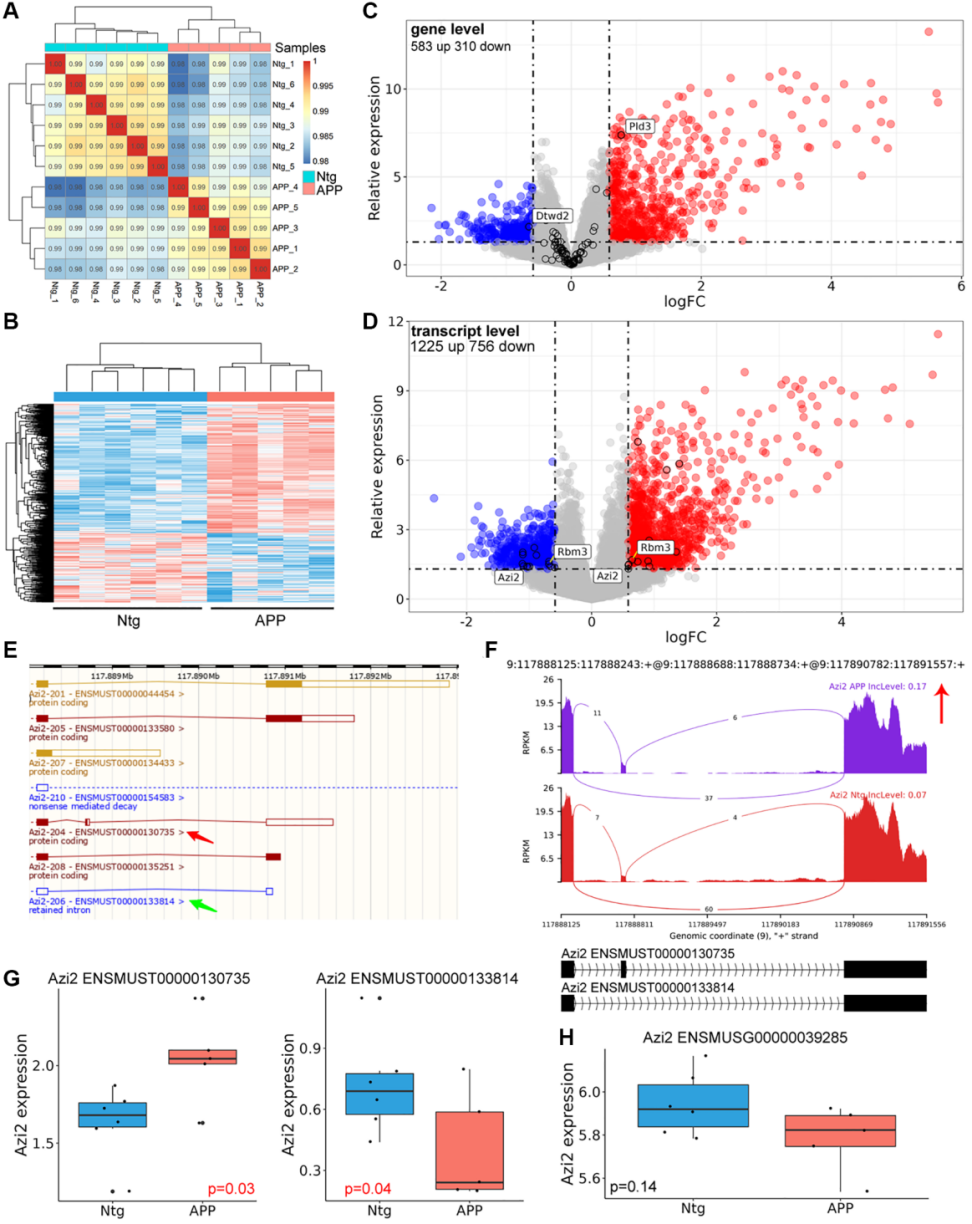
**Differentially genes and transcripts analysis.** (**A**) Correlation analysis with gene expression matrix. (**B**) Heatmap of dysregulated genes between APP and Ntg group. (**C**) Volcano plot of dysregulated genes. The black circle indicated genes with significant AS event. (**D**) Volcano plot of dysregulated transcripts. Genes harbored different transcripts with opposite changes were labeled. (**E**) Genomic information of Azi2. Red arrow indicated the longer transcript with coding ability and SE event, and the green arrow revealed the shorter transcript with RI. (**F**) The detailed sashimi plots for Azi2. (**G**, **H**) Relative expression of Azi2 in gene and transcript levels. ENSMUST00000130735 represented longer Azi2 transcript, and ENSMUST00000133814 represented the shorter transcript.

Aberrantly spliced mRNAs would be degraded by nonsense-mediated mRNA decay (NMD) pathway. However, some mRNAs may bypass NMD and be translated. Differential expressed genes and transcripts were examined. These samples were well correlated within group (Figure 5A). 583 up- and 310 down-regulated genes were identified (Figure 5B, Supplementary Table 2). Only two genes with significant AS events showed significant difference between APP and Ntg groups on gene level (Figure 5C). On transcript level, 1225 up- and 756 down-regulated transcripts were identified (Supplementary Table 3). 17 out of the 26 transcripts, whose host genes with significant AS events, were involved in SE event, 6 transcripts were involved in RI event (Table 3). Several genes with significant AS events harbored transcripts with opposite changes, including Rbm3 and Azi2 (Figure 5D, Table 3). Given Azi2 for instance, SE event resulted in a longer transcript (ENSMUST00000130735) and a shorter transcript (ENSMUST00000133814) (Figure 5E). In SE event, Azi2 showed increased PSI for the longer transcript in APP group (Figure 5F). Consistently, the longer ENSMUST00000130735 with increased PSI in APP group was elevated, while the shorter ENSMUST00000133814 with decreased PSI in APP group showed suppressed expression (Figure 5G). However, the gene level expression of Azi2 showed no significant difference (Figure 5H).

**Table 3:** Dysregulated transcripts involved in significant AS genes.

**ID**	**Gene**	**Transcript**	**logFC**	***P*.Value**	**Change**	**AS type**
3492	Il4i1	ENSMUST00000033015	1.402153	1.43E-06	UP	SE
7070	Lrrc56	ENSMUST00000070458	0.738023	0.024408	UP	SE
11659	Rbm3	ENSMUST00000115621	0.647311	0.020246	UP	RI
11784	Pld3	ENSMUST00000117095	0.739458	1.60E-07	UP	MXE
13865	Eme2	ENSMUST00000128953	−0.91856	0.006028	DOWN	RI
14316	Azi2	ENSMUST00000130735	0.585534	0.034119	UP	SE
14853	Nfat5	ENSMUST00000133026	−0.68301	0.02389	DOWN	SE
15031	Azi2	ENSMUST00000133814	−0.99508	0.039242	DOWN	SE
15298	Rnf135	ENSMUST00000134909	0.923443	0.038781	UP	SE
15611	Arhgef11	ENSMUST00000136072	−1.0361	0.038672	DOWN	SE
16477	Il15ra	ENSMUST00000139774	1.351781	0.009183	UP	SE
16644	St7l	ENSMUST00000140457	−1.02025	0.041252	DOWN	SE
17009	Rbm3	ENSMUST00000141925	−0.68297	0.0309	DOWN	RI
20175	Pld3	ENSMUST00000155287	1.200779	2.63E-06	UP	MXE
20796	Adck5	ENSMUST00000159949	−1.10756	0.029743	DOWN	RI
21734	Tmem176b	ENSMUST00000164733	0.594122	0.031092	UP	SE
22481	Ankrd13d	ENSMUST00000170283	−1.09135	0.03766	DOWN	SE
23261	Pknox1	ENSMUST00000175806	1.252792	0.006284	UP	SE
24619	2410002F23Rik	ENSMUST00000188111	0.920056	0.002927	UP	SE
26213	Krit1	ENSMUST00000200386	−0.59063	0.043742	DOWN	A3SS
26680	Tmem176b	ENSMUST00000203229	0.587406	0.049277	UP	SE
26995	Emsy	ENSMUST00000205331	−0.88422	0.012687	DOWN	SE
27595	Tpcn2	ENSMUST00000209047	−1.10109	0.011942	DOWN	SE
27876	Wdr74	ENSMUST00000210592	0.903649	0.02283	UP	RI
30538	Camk2g	ENSMUST00000225958	−0.65708	0.038834	DOWN	SE
32057	Sema4g	ENSMUST00000235312	−1.10267	0.009556	DOWN	RI

### Analysis of upstream splicing factors

**Figure 6:**
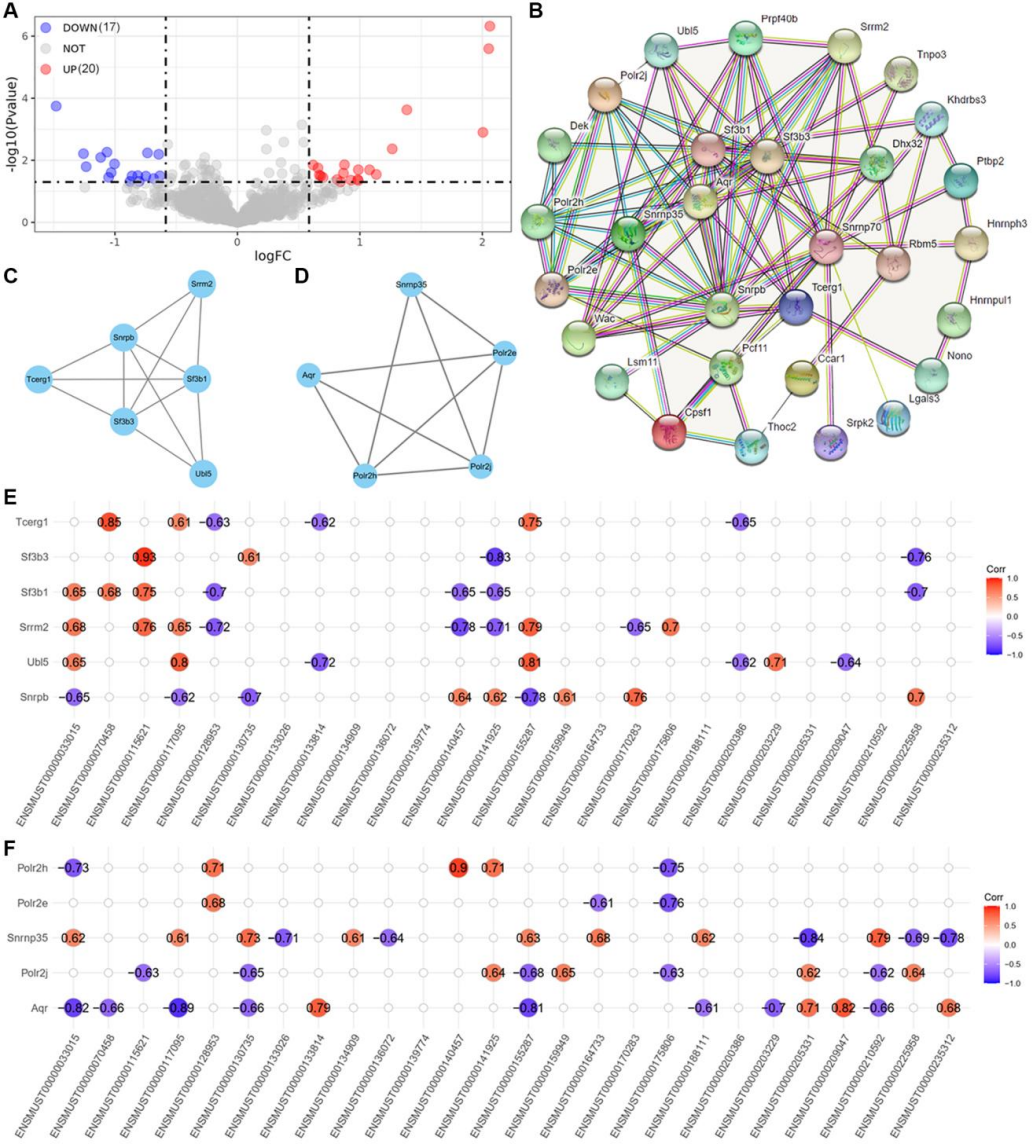
**Analysis of upstream splicing factors.** (**A**) volcano plot of dysregulated splicing factors. (**B**) PPI networks of these 37 dysregulated splicing factors. (**C**, **D**) The first and second cluster of these splicing factors selected using MCODE app in Cytoscape. (**E**) Correlation between the firster cluster splicing factors and dysregulated transcripts involved in significant AS events. (**F**) Correlation between the second cluster splicing factors and dysregulated transcripts involved in significant AS events.

AS events could be regulated by a number of splicing factors (SFs). The expression of 280 SFs obtained from previous study was examined (Supplementary Table 4) [12]. A total of 37 transcripts involved 30 SF genes were significantly dysregulated between APP and Ntg groups (Figure 6A, Table 4). The PPI networks were constructed using String and Cytoscape (Figure 6B). The first and second clusters of these SFs were identified using Cytoscape with MCODE app (Figure 6C, 6D). The correlation of the two clusters and significantly dysregulated transcripts whose host genes were involved in significant AS events were performed (Figure 6E, 6F).

**Table 4:** Dysregulated transcripts of splicing factors.

**Gene**	**Transcript**	**logFC**	***P*.Value**	**Change**
DHX32	ENSMUST00000063669	1.383375	0.000237	UP
AQR	ENSMUST00000102543	−1.4803	0.000179	DOWN
POLR2J	ENSMUST00000111127	−1.11641	0.008073	DOWN
SNRNP35	ENSMUST00000111453	2.004711	0.001256	UP
HNRNPH3	ENSMUST00000119814	0.986423	0.045196	UP
LSM11	ENSMUST00000129820	−0.63295	0.031731	DOWN
POLR2E	ENSMUST00000131743	−0.81305	0.031261	DOWN
DEK	ENSMUST00000138401	0.865551	0.025996	UP
LGALS3	ENSMUST00000146468	2.061378	4.77E-07	UP
NONO	ENSMUST00000148312	0.842566	0.049619	UP
PRPF40B	ENSMUST00000150641	−0.68416	0.03879	DOWN
SNRPB	ENSMUST00000150835	−0.74767	0.032351	DOWN
PCF11	ENSMUST00000151177	0.813964	0.041743	UP
THOC2	ENSMUST00000151454	−1.25672	0.00612	DOWN
POLR2H	ENSMUST00000161038	−1.031	0.024872	DOWN
UBL5	ENSMUST00000161882	1.078713	0.020229	UP
TNPO3	ENSMUST00000164325	−0.80254	0.047883	DOWN
WAC	ENSMUST00000171042	−1.05179	0.035422	DOWN
RBM5	ENSMUST00000182304	−0.73468	0.005779	DOWN
SRRM2	ENSMUST00000186045	0.618158	0.014162	UP
SF3B1	ENSMUST00000189051	0.699037	0.035869	UP
PTBP2	ENSMUST00000196343	−1.06551	0.005463	DOWN
SRPK2	ENSMUST00000197622	0.68085	0.031996	UP
HNRNPUL1	ENSMUST00000206260	−0.87732	0.047941	DOWN
SNRNP70	ENSMUST00000209858	−1.00368	0.013086	DOWN
SF3B3	ENSMUST00000212515	0.926118	0.043761	UP
CCAR1	ENSMUST00000218437	0.668309	0.029909	UP
CPSF1	ENSMUST00000230822	0.982423	0.041531	UP
KHDRBS3	ENSMUST00000230847	−0.64257	0.006404	DOWN
TCERG1	ENSMUST00000237602	0.658058	0.017652	UP

## DISCUSSION

Aβ plaque is a hallmark of AD. Aβ is produced by sequential cleavage of APP, and APP transgenic mice have been widely used ad AD model [13, 14]. In this study we explored the AS events in hippocampal microglia sorted by CD45 and CD11b in APP and Ntg mice. All five types of AS events were detected, and genes involved different AS events showed AS type specific GO enrichment. Aberrant AS have been widely recorded in AD patients and animal models [10, 11]. We for the first time, investigated cell type specific AS events in AD.

Consistent with previous studies [15, 16], SE was the most abundant AS events (68.42%), leading to diverse protein isoforms encoded by a single gene. A3SS (11.58%) and RI (10.53%) showed similar AS event counts. GO analysis of genes involved in significant SE events were enriched in regulation of autophagy (Figure 2A, 2B), including Eif2ak4 and Tpcn2. Eif2ak4 phosphorylates Eif2a in response to various stress stimuli including amino acid starvation. Phosphorylation of Eif2a subsequently promotes translation of activating transcription factor 4 (Atf4). Atf4 is a key autophagy related gene. Atf4 binds to promoters of numerous autophagy genes including Becn1, Atg7, and Sqstm1, and this subsequently increased transcription of these genes. Therefore, activation of Eif2ak4-Eif2a-Atf4 pathway triggers autophagy [17]. Here, we found that Eif2ak4 tended to generate a longer transcript in AD, the function of this longer isoform may deserve further functional studies. Tpcn2 is a lysosomal non-selective Na^+^/Ca^2+^ channel, Tpcn2 suppresses the fusion between autophagosome and lysosome, leading to the accumulation of autophagosomes in cancer cells [18]. In this study, we found that Tpcn2 tended to produce a shorter transcript in AD.

A3SS occurred due to alternative acceptor sites, and the PSI indicates inclusion of the longer exon in A3SS events. GO enrichment revealed that genes with significant A3SS events were enriched in actin polymerization and depolymerization, including Mical1 and Add1. Tau accumulation is clearly linked to AD pathogenesis. Mical1 was identified as tau interacting protein via proteomic approach. Mical1 changed the interaction properties of tau and potentiated tau toxicity, mediated by Mical1’s redox activity on tau Cys322 [19]. Moreover, the protein level of Mical1 was increased in AD patients, and considered to be a potential biomarker of tauopathies [19]. Add1 plays essential role in dendritic morphology. Zheng et al. reported that loss of miR-135a-5p expression increased Rock2 activity and phosphorylation of Add1 at Ser726, leading to dendritic abnormalities and memory impairments in AD [20].

Most mRNA derived from RI event may be degraded via NMD, however, some mRNAs could still be translated [11]. Here, we found that genes spliced by RI were enriched in synaptic plasticity including axonogenesis, neurotransmitter secretion, neuron projection and axon guidance (Figure 4A, 4B). Gpc2 tended to harbor an intron in APP group (Figure 4C). Gpc2 has been considered as novel AD risk genes which determine the microglia response to Aβ but not to tau pathology [21].

Moreover, we found that AS events mainly cause dysregulation of genes in transcript level rather than gene expression level (Figure 5C, 5D). Among the 90 genes involved in significant AS event, only 2 genes (Pld3, Dtwd2) were remarkably dysregulated on gene level. However, on transcript level, 26 transcripts refer to 22 genes involved in significant AS events. Rbm3 and Azi2 harbored transcripts with opposite changes in AD. In addition, AS event level was consistent with corresponding transcripts in Azi2 genes (Figure 5E–5G), without effect on gene level (Figure 5H). Moreover, 17 out of the 26 transcripts, whose host genes with significant AS events, were involved in SE event, 6 transcripts were involved in RI event (Table 3). These results suggest that SE and RI events may lead to transcript level dysregulation of different genes in hippocampal microglia in AD. We have also performed GO enrichment analysis of the 17 transcripts with SE event, it seems that these transcripts were not associated with the phenotypes of hippocampal microglia (data were not shown).

The upstream SFs were also explored. 37 significantly dysregulated SF transcripts refer to 30 SFs were identified (Figure 6A). Two main clusters were selected from SFs PPI networks, and correlation analysis was performed between these SFs and transcripts involved in AS events. Several SFs showed significantly positive or negative correlation with these transcripts (Figure 6E, 6F). These results suggest that dysregulated SFs may govern the AS events of critical AD pathogenesis related genes described here. We have searched several splicing factor databases to explore AS type-specific preferences of these SFs, including SpliceAid-F (http://www.caspur.it/SpliceAidF/), SpliceAid 2 (http://193.206.120.249/splicing_tissue.html), RBPmap, and MiasDB (http://47.88.84.236/Miasdb/index.php). However, SFs preferences for different five basic modes of AS have not been identified. AS type-specific preferences of SFs need further investigation.

In conclusion, we described hippocampal microglia AS in AD. Autophagy related genes were mainly spliced in SE events, actin depolymerization genes were spliced in A3SS events, while synaptic plasticity related genes were mainly spliced in RI pattern. Dysregulation of autophagy, actin depolymerization, and synaptic plasticity have been widely documented in AD. Our results provide a novel therapeutic direction based on AS regulation. These significant AS events may be regulated by dysregulated splicing factors in AD. Our study provides novel pathological mechanisms in the pathogenesis of AD.

## MATERIALS AND METHODS

### Dataset and reference genome

GSE171195 was deposited in GEO (https://www.ncbi.nlm.nih.gov/geo/query/acc.cgi?acc=GSE171195), and the raw data were downloaded from ENA database (https://www.ebi.ac.uk/ena/browser/view/PRJNA718619) using axel (v2.17.5) and checked using md5sum (v8.30). GSE171195 performed RNA sequencing of hippocampal microglia cells from APP and Ntg mice. The gtf, cDNA fasta, and DNA fasta files were downloaded from ENSEMBL database as reference.

### Reads alignment

FastQC (v0.11.9) and multiqc (v1.11) were used for quality control. Trim-galore (v0.6.7) was used for adaptors and low-quality trimming with parameters “trim_galore --phred33 -q 20 --length 36 --stringency 3 --fastqc --max_n 3”. STAR (v 2.7.10a) was used to align the clean data to the reference genome downloaded from ENSEMBL [22], with parameters “STAR --runThreadN 6 --readFilesCommand zcat --outSAMtype BAM SortedByCoordinate --outBAMsortingThreadN 6”.

### Gene/transcript expression matrix

The aligned and sorted bam files were proceeded to generate gene expression matrix with featureCounts (v2.0.1) [23], with parameters “featureCounts -T 8 -p -t exon -g gene_id”. Clean fastq files after data trimming were used to generate transcript expression matrix with salmon (v1.0.4) [24].

### Differential expressed gene/transcript analysis

The gene and transcript expression matrices were used for differential expression analysis using limma R package (v3.50.0) [25]. The cutoff values for *p* value and logFC were 0.5 and 1.5 respectively.

### Alternative splicing analysis

The sorted bam files were used for AS analysis with rMATS (v4.1.2) [26] and plotted using rmats2sashimiplot (v2.0.4), with parameters “rmats.py --b1. /b1.txt --b2. /b2.txt --gtf. /Mus_musculus.GRCm39.105.gtf -t single --readLength 100 --nthread 30 --od. /output --tmp. /tmp_output”. Significant AS events were considered if average coverage >5 and delta percent spliced in (ΔPSI) >0.1. Statistics of AS events was performed using maser (v1.12.1) R package. The code for the above methods were similar with our previous codes deposited Github (https://github.com/tjhwangxiong/AD-GSE132177-Alternative-Splicing-pipeline).

### Gene ontology enrichment analysis

Gene ontology (GO) enrichment analysis was performed with clusterProfiler (v4.2.2) [27], and GOplot (v1.0.2) [28] R packages. The *p* value < 0.05 was considered as significant GO terms.

### Protein-protein interaction network analysis

The protein-protein interaction (PPI) network analysis was performed using String (https://cn.string-db.org/) and Cytoscape (v3.8.2) [29]. The MCODE app was used to build sub clusters with default settings. The first and second clusters of significantly dysregulated splicing factor transcripts were selected for further transcript correlation analysis.

### Transcript correlation analysis

The correlation between significantly dysregulated splicing factor transcripts and significantly dysregulated transcripts whose host genes harbored significant AS events was performed using ggcorrplot R package (v0.1.3). The correlation and *p* values were calculated. Only correlation results with *p* value < 0.05 were presented.

### Data availability statement

The GSE171195 dataset was deposited in GEO database: https://www.ncbi.nlm.nih.gov/geo/query/acc.cgi?acc=GSE171195. Other data were included in the article or supplementary files.

## Supplementary Materials

Supplementary Table 1

Supplementary Table 2

Supplementary Table 3

Supplementary Table 4
